# Supplementary material for: dubTAGs enable on-demand stabilization for tunable and reversible control of endogenous protein levels
Source: bioRxiv. 2026 Sep 18:2026.09.11.751045. Preprint. [Version 1] doi: 10.64898/2026.09.11.751045 (PMC13596203; doi:10.64898/2026.09.11.751045)
Supplement: Supplementary file 1 [file NIHPP2026.09.11.751045v1-supplement-1.pdf]

bioRxiv preprint doi: https://doi.org/10.64898/2026.09.11.751045; this version posted September 18, 2026. The copyright holder for this preprint (which was not certified by peer review) is the author/funder, who has granted bioRxiv a license to display the preprint in perpetuity. It is made available under a CC-BY 4.0 International license.

Supplementary Figures

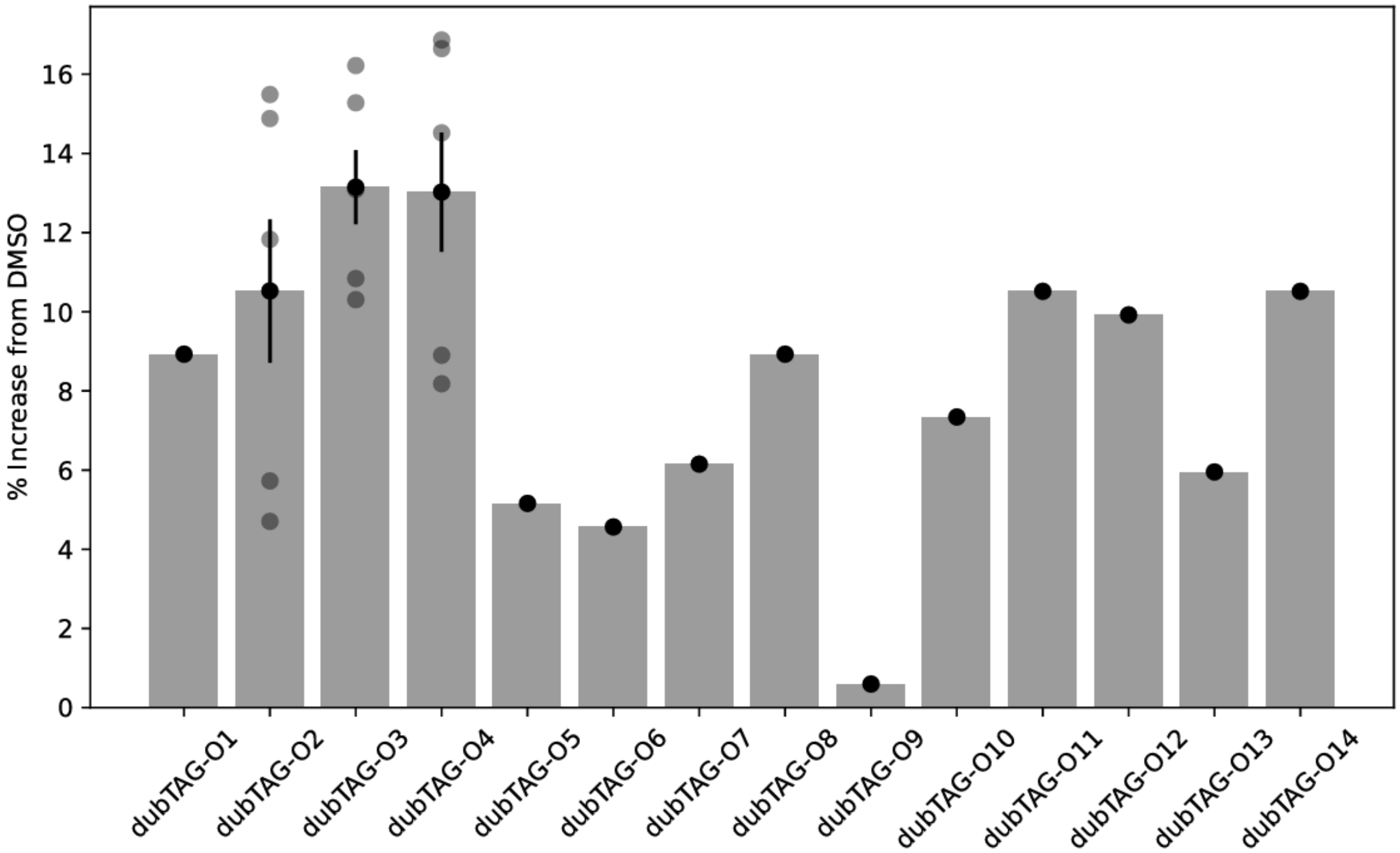

Figure S1. Initial screen of dubTAG effects on SOX9 levels in hESCs. Data are plotted as a percentage increase from untreated levels. All compounds were applied at 1 uM for 48h. Light points represent independent biological replicates, dark point and error bars indicate mean and standard error. Data from compounds with one biological replicate are shown for completeness and were not characterized further.

bioRxiv preprint doi: https://doi.org/10.64898/2026.09.11.751045; this version posted September 18, 2026. The copyright holder for this preprint (which was not certified by peer review) is the author/funder, who has granted bioRxiv a license to display the preprint in perpetuity. It is made available under a CC-BY 4.0 International license.

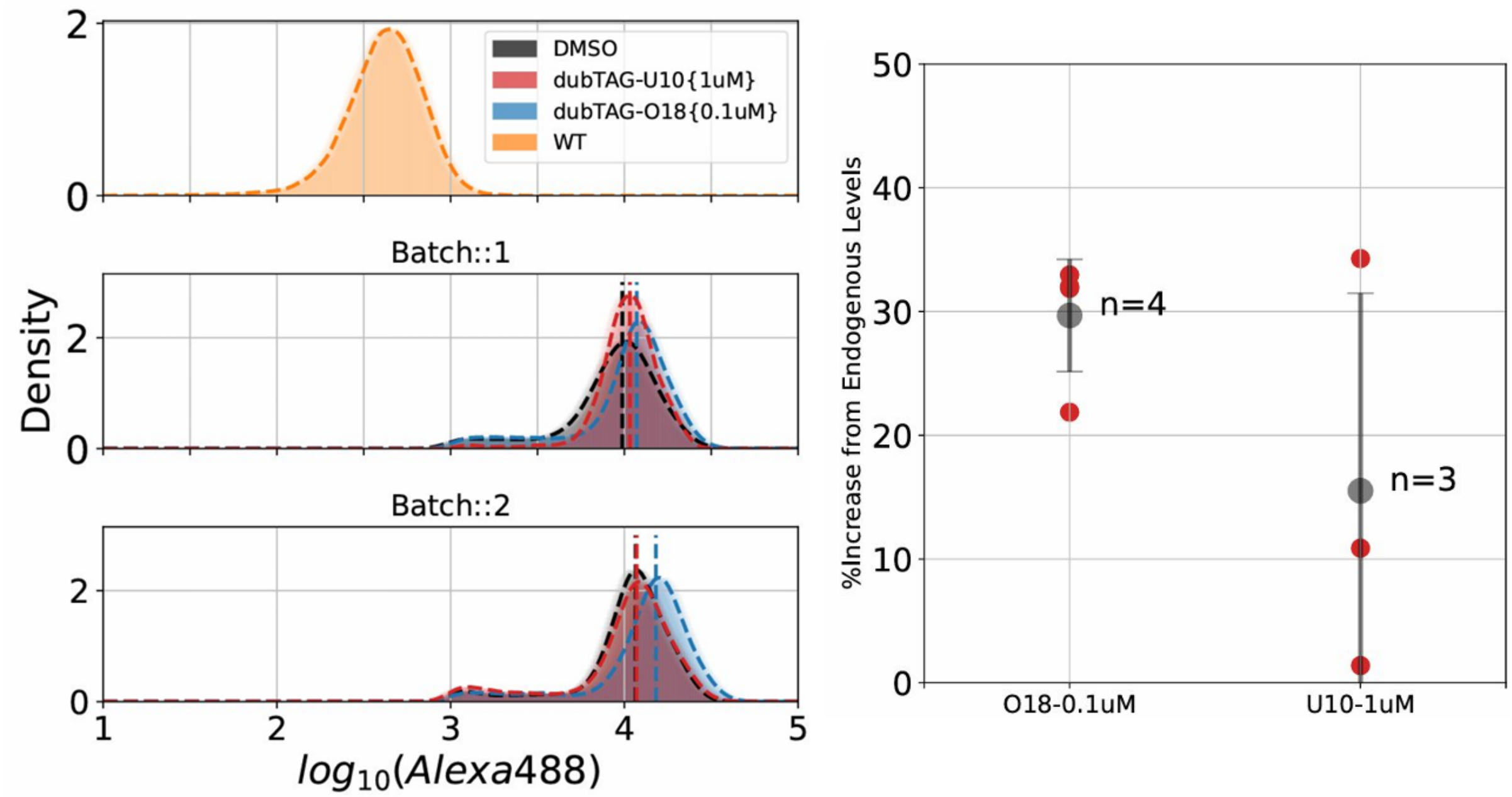

Figure S2. Selected dubTAGs boost endogenous levels of FKBP12^{F36V} tagged TWIST1 in CNCCs. Treatment of TWIST1-FKBP12^{F36V}-V5 edited CNCCs with dubTAG-O18 and dubTAG-U10 at maximal effective concentrations as evaluated in SOX9--FKBP12^{F36V} edited CNCCs. (Left Panel) Intracellular flow-cytometry measurements across treatment conditions from two representative CNCC differentiation batches derived from TWIST1 edited h9 ESCs. (Right Panel) Quantification of TWIST1 increases from endogenous levels from dubTAG treatments. The number of replicates denote distinct differentiation batches.

bioRxiv preprint doi: https://doi.org/10.64898/2026.09.11.751045; this version posted September 18, 2026. The copyright holder for this preprint (which was not certified by peer review) is the author/funder, who has granted bioRxiv a license to display the preprint in perpetuity. It is made available under a CC-BY 4.0 International license.

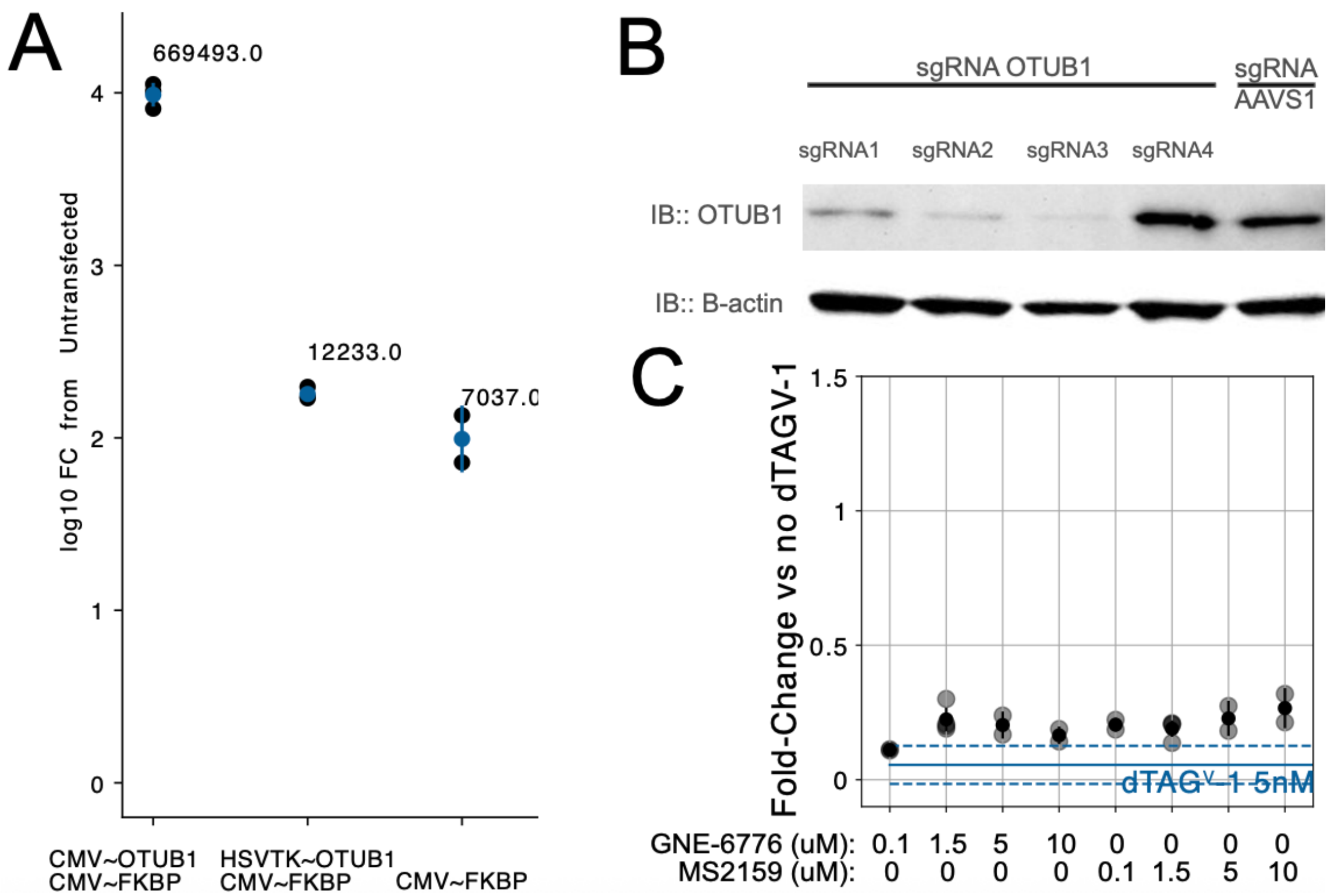

Figure S3. Supplementary figure related to Figure 3. (A) Change in luciferase signal (y-axis, log10 scale) relative to untransfected cells in untreated, OTUB1 WT HEK293 cells transfected with the indicated plasmids. Numbers on top of each point indicate the mean raw luciferase signal in each condition. (B) Immunoblot of HEK293 cells transfected with plasmids encoding the indicated sgRNAs targeting OTUB1 or AAVS1 (control). (C) Effect of indicated deubiquitinase binders (all 24h) on SOX9 levels following 24h 5nM dTAG^{V}-1 pretreatment (of which mean and is standard error indicated by blue solid and dashed lines, respectively). Light black points represent independent biological replicates, dark points and error bars indicate mean and standard error.

bioRxiv preprint doi: https://doi.org/10.64898/2026.09.11.751045; this version posted September 18, 2026. The copyright holder for this preprint (which was not certified by peer review) is the author/funder, who has granted bioRxiv a license to display the preprint in perpetuity. It is made available under a CC-BY 4.0 International license.

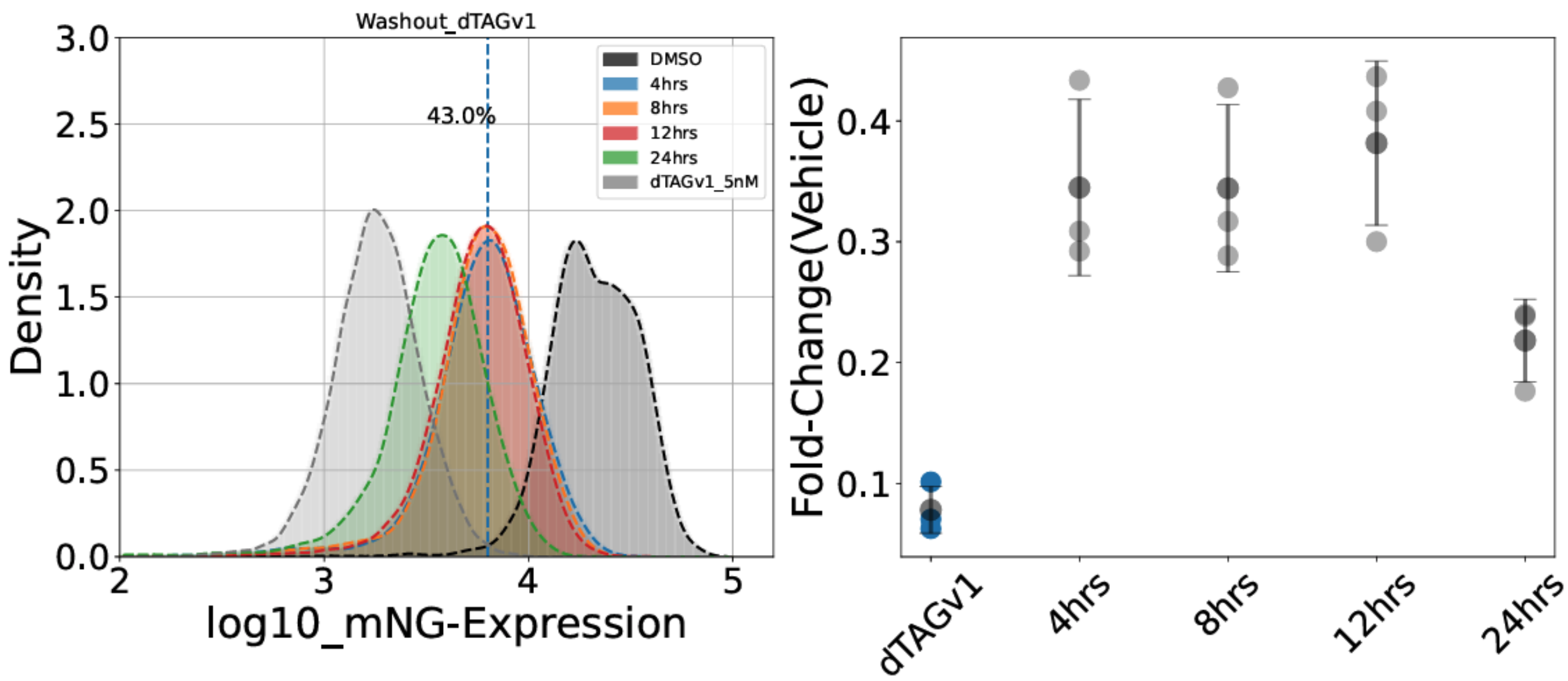

Figure S4. Washout of dTAG^{V}-1 does not fully restore SOX9 levels. Left, representative flow cytometry histogram of mNeonGreen (SOX9) levels at distinct timepoints (colors) following washout of 5 nM dTAGV-1. Right, quantification of SOX9 level (median of flow cytometry distributions) at indicated timepoints (x-axis) following washout. Light points represent independent biological replicates, dark point and error bars indicate mean and standard error.

bioRxiv preprint doi: https://doi.org/10.64898/2026.09.11.751045; this version posted September 18, 2026. The copyright holder for this preprint (which was not certified by peer review) is the author/funder, who has granted bioRxiv a license to display the preprint in perpetuity. It is made available under a CC-BY 4.0 International license.

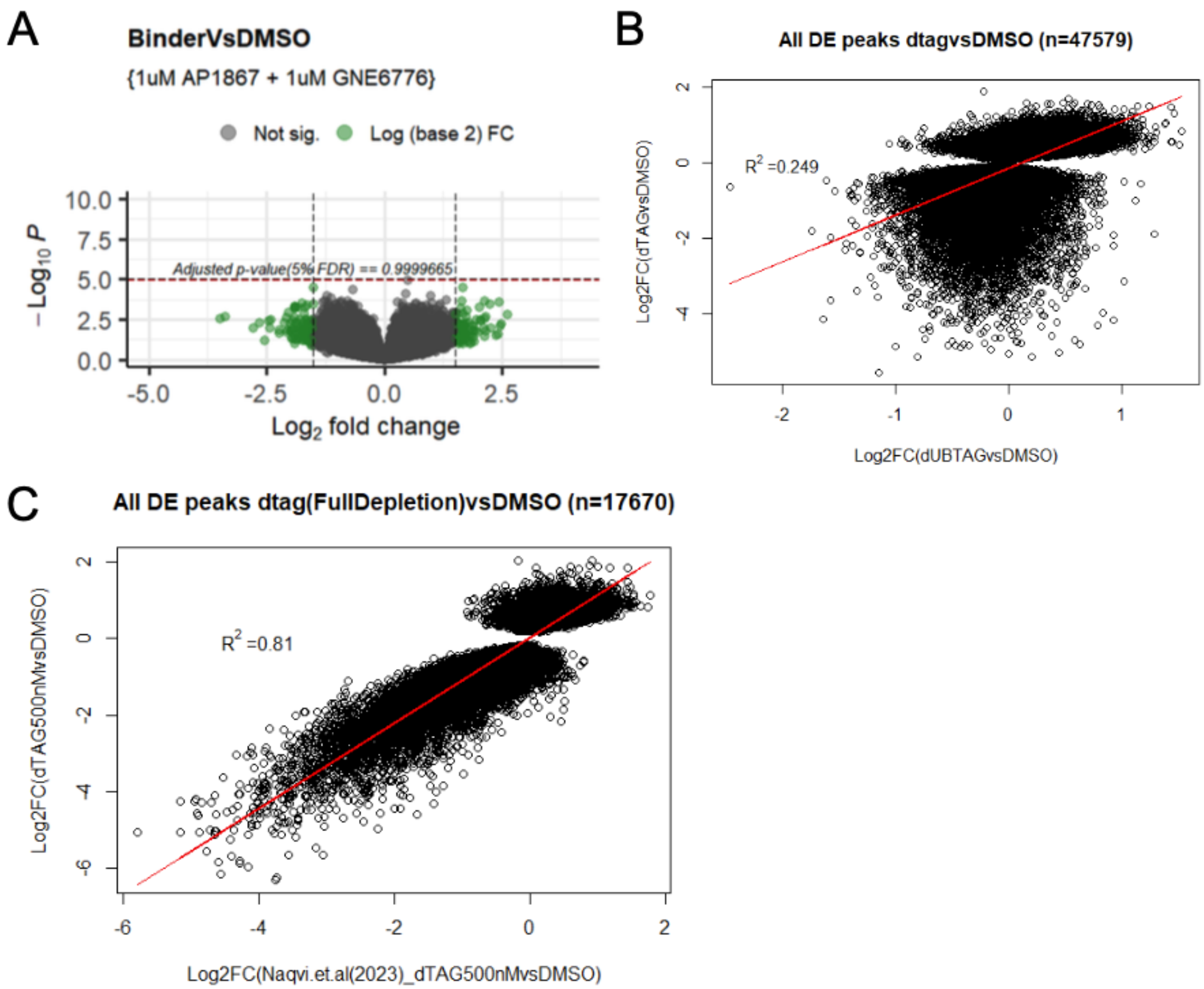

Figure S5. Analyses related to effects of increased SOX9 levels on chromatin accessibility in CNCCs. (A) Volcano plot of differential accessibility analysis comparing AP1867 and GNE-6776 binder treatment to DMSO. (B) Comparison of dubTAG-U10 treatment (x-axis) effect versus full SOX9 depletion effect (500 nM $dTAG^{V}$-1) (y-axis) from this study. (C) Comparison of full SOX9 depletion effect between this study (y-axis) and Naqvi et al 2023 (x-axis).

bioRxiv preprint doi: https://doi.org/10.64898/2026.09.11.751045; this version posted September 18, 2026. The copyright holder for this preprint (which was not certified by peer review) is the author/funder, who has granted bioRxiv a license to display the preprint in perpetuity. It is made available under a CC-BY 4.0 International license.

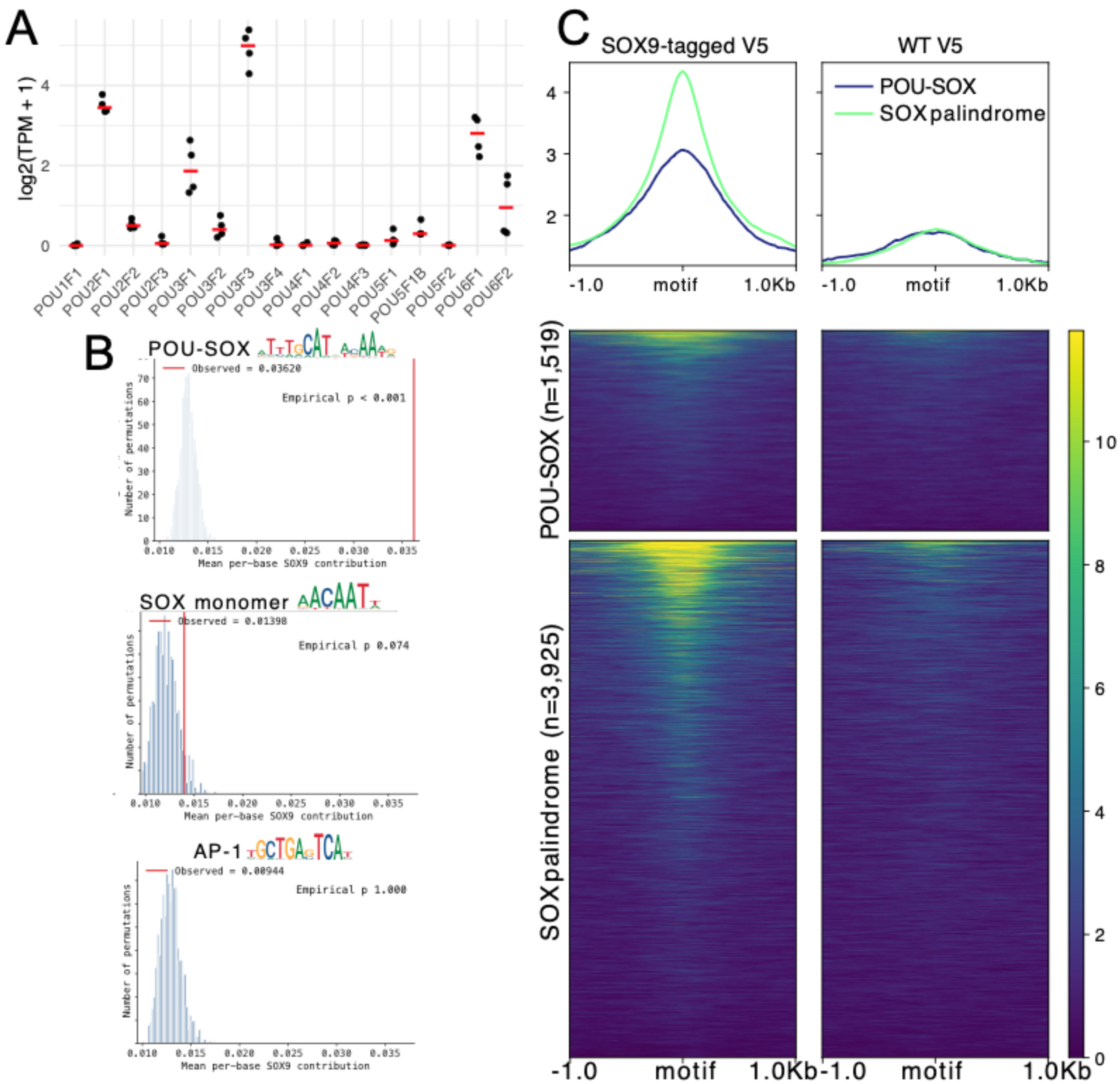

Figure S6. POU-SOX motif instances likely function as low-affinity SOX9 binding sites. (A) Transcripts per million (TPM) of indicated POU family TFs (x-axis) in unperturbed CNCCs (data from Naqvi et al 2025). Points represent individual biological replicates, red horizontal line represents mean. (B) Mean per-base contribution of the indicated motifs (red line), as quantified by a BPNet model trained on CNCC SOX9 (V5 ChIP-seq), compared to 1,000 permutations where motif positions were shuffled within the same REs. (C) V5 ChIP-seq signal from CNCCs with V5-tagged SOX9 present ('SOX9-tagged V5') or absent ('WT') plotted at regions centered around POU-SOX (top) or SOX9 palindrome (bottom) instances within accessible regions.
